# Large-scale production of the malaria vector biocontrol agent *Romanomermis iyengari* (Nematoda: Mermithidae) in Benin, West Africa

**DOI:** 10.5281/zenodo.10869973

**Published:** 2015-01-17

**Authors:** Thiery BC Alavo, Ayaba Z Abagli, Rafael Pérez-Pacheco, Edward G Platzer

**Affiliations:** 1 Laboratoire d’Entomologie appliquée/Centre Edward Platzer, Université d’Abomey-Calavi, BP 215 Godomey, Benin; 2 CIIDIR Oaxaca, Instituto Politécnico Nacional, Xoxocotlan, Oaxaca, C.P.71230, Mexico; 3 Department of Nematology, University of California, Riverside, CA 92521-0415, USA

## Abstract

**Background:**

The mermithid nematode *Romanomermis iyengari* is one of several natural control alternatives to synthetic pesticides for mosquito suppression. The commonly used mass rearing procedure of *R. iyengari* involves the use of coarse sand as a substrate for nematode maturation and oviposition. The coarse sand technique gives excellent nematode productivity in North America. However, under West African climatic conditions, this technique generates relatively lesser amounts of infectious worms. We evaluated coconut coir fibres as a replacement for coarse sand to improve yields in large-scale production of *R. iyengari* in Benin, West Africa.

**Materials and Methods:**

*Culex quinquefasciatus* was the host for the nematodes, and mosquitoes were blood-fed on chickens. Four days after blood feeding, egg rafts were collected and transferred into trays, each containing 2 l of water. The mosquito larvae were fed with fish food. When the mosquito larvae reached the second instar, preparasites (J2) were added (3 J2/larva) to the incubation trays. Eight days after infection, post-parasitic juveniles were separated from the water containing dead mosquito larvae and other debris using sieves and needles; 2 g of them were deposited in containers with coarse sand or coconut coir fibres and water. Three hours later, the water was drained, the jars covered and stored for eight weeks, after which J2 abundance was determined, using a total of 320 containers for each substrate. The abundance of J2 preparasites was also assessed 3-5 months after storage to determine the impact of long-term storage on the J2 yield.

**Results:**

After 2 months storage, 2 g of post-parasites (~457 females and 583 males) yielded an average of 559,300±6094 J2 and 155,818±4427 J2 per container for coconut fibres and for coarse sand, respectively. During long-term storage, yields of J2 on coconut fibres substrate slowly decreased from 442,180±9322 J2 (3 months storage) to 163,632±12,416 J2 per container (5 months storage). On coarse sand substrate, the yield was relatively low and decreased from 49,812±1200 J2 at 3 months storage to 3046±229 J2 at 5 months storage.

**Conclusion:**

Under West African climatic conditions, coconut coir fibres gave significantly higher preparasitic nematode yields than the coarse sand technique.

## 1 Introduction

The intensive use of chemical insecticides against mosquitoes has led to the development of widespread insecticide resistance in Benin [1]. There is an urgent need for malaria control programmes to adopt more integrated mosquito management approaches that include sustainable, non-chemical solutions. The mermithid nematode *Romanomermis iyengari* Welch is one of several natural control alternatives to synthetic pesticides for mosquito suppression. This parasite of mosquito larvae was first reported in 1927 in the Lower Bengal Delta (India) in *Anopheles* and *Culex* larvae [2,3]. It develops inside mosquito larvae that die when the parasite emerges [4]. Platzer [5] summarised multiple characteristics of mermithids that make them attractive for biological control of mosquitoes, including ease of application, environmental safety, host specificity, laboratory manipulation of life history, lethality, mass rearing *in vivo* and their potential for long-term recycling in the environment.

Several research groups have demonstrated the effectiveness of *R. iyengari* for mosquito control in North and Latin American countries, as well as in India and several other countries [2,4,6-12]. Moreover, the use of this biocontrol agent for malaria vector control has induced a rapid and progressive decrease of malaria prevalence in Colombia [13]. Surprisingly, despite the usefulness of *R. iyengari* in malaria control, until 2011, this biocontrol agent had not been tried for vector control in Africa, where there are still 174 million annual malaria cases [14].

Recently, research conducted in Benin using samples of *R. iyengari* produced in California/USA, has shown the efficacy of this nematode for the control of *Anopheles gambiae s.l.*, a major malaria vector in sub-Saharan Africa. Tests conducted in the laboratory with this nematode against anophelines revealed that this nematode parasitises and kills larvae within days. Furthermore, monthly applications of this nematode in several natural mosquito breeding sites in southern Benin resulted in suppression of larval development [15,16]. Subsequent to this success, a rearing centre was constructed in Benin to produce large amounts of *R. iyengari* for use against malaria vectors. This centre used the large-scale production technique developed for mermithids in the USA and Mexico [17-19]. This rearing procedure involves the use of coarse sand for nematode incubation to allow post-parasitic worms to oviposit normally. This coarse sand technique yields excellent nematode productivity in North America. Nevertheless, under West African climatic conditions, this technique generates relatively less amounts of infectious worms [20]. Moreover, coarse sand is heavy, and during transportation from the rearing centre to the sites for larval control intervention, the movements of sand particles can also crush the fragile nematodes and eggs [21-23].

Here we studied the use of coconut coir fibres (decorticated husk) instead of coarse sand for large-scale production of the mermithid nematode *R. iyengari* in Benin. Yields of pre-parasitic nematodes obtained with coarse sand were compared with those using coconut coir fibres.

## 2 Materials and Methods

### 2.1 Mosquito breeding

Culex *quinquefasciatus* was used as host for the nematodes. Chickens (*Gallus domesticus*) were used to supply these mosquitoes with blood meals. Mosquito larvae were fed every 3 days with fish food and the adult mosquitoes were permanently supplied with 10% sugar water by means of cotton soaked in the sugar solution in an Erlenmeyer flask. The insectary generates up to 500,000 larval mosquitoes weekly. Temperature and relative air humidity in the insectary were 28±2°C and 70-90% RH, respectively.

### 2.2 Rearing of *R. iyengari* nematodes

**Figure 1.**
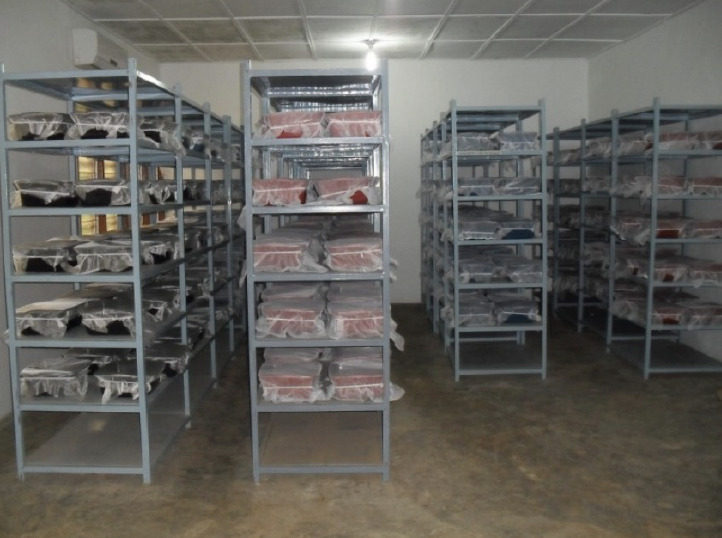
Nematode production room

**Figure 2.**
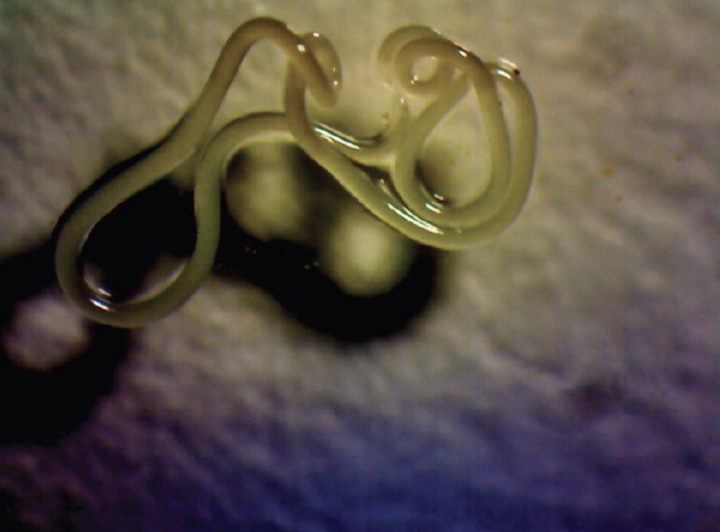
Post-parasitic *R. iyengari* (20× magnification)

A small plastic tray (12×8×6 cm) containing about 500 ml of water was provided in each mosquito cage for oviposition. Four days after a blood meal the egg rafts, which contained about 120 eggs each, were collected and 6 of these were deposited in a plastic tray (25×15×12 cm) containing 2 l water. Five hundred plastic trays were installed on metal shelves in the nematode production room (Figure 1) where temperature and relative humidity were 28±2°C and 70-90% RH, respectively. The containers were covered with a mesh screen to prevent oviposition by wild mosquitoes. When the larvae reached the second instar (2 days), they were infected with preparasitic nematodes (second stage juveniles, J2) of *R. iyengari*, by the addition of 3 J2 per mosquito larva. The nematodes were taken from cultures that had been stored for eight weeks. These cultures were flooded with sterilised (chlorine-free) water to induce the eclosion of eggs and emergence of infective preparasites from the substrate. Fourteen hours after flooding the cultures (overnight), the water was decanted, and the concentration of nematodes in the solution calculated by volumetric dilution [18]. After 8 days, the mosquito larvae died and floated on the surface, indicating the end of the parasitic phase of the nematode. The water containing post-parasitic juveniles (J4) together with dead mosquito larvae and other debris was poured onto a sieve. The sieve containing the larvae and J4 was then placed in a container with clean water. After a few minutes, the J4 passed through the sieve into the clean water so that when the sieve was removed, the dead mosquito larvae and debris were separated from the J4 that settled on the bottom of the container. The J4 (Figure 2) were collected using a syringe and transferred to a glass beaker with clean water. The J4 were then washed thoroughly several times by sedimentation.

**Figure 3.**
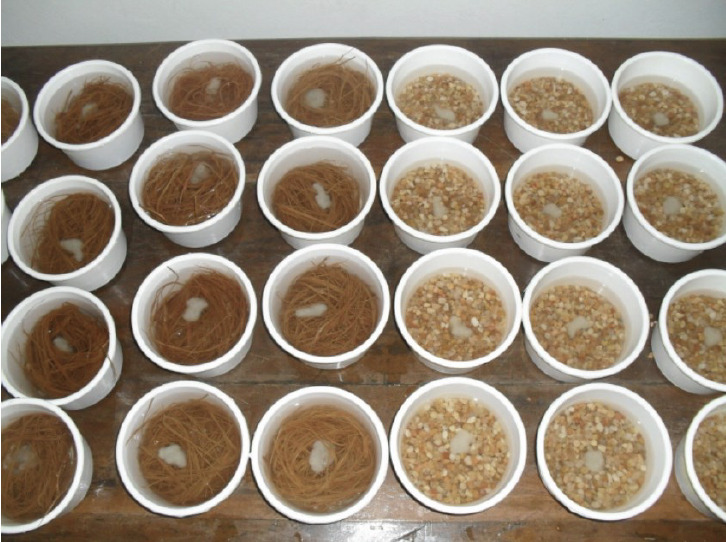
Post-parasitic nematodes on coconut coir fibres (containers on the left) and on coarse sand (containers on the right)

Two grams (wet weight) of washed J4 (composed of 457.6±1.38 females and 583±0.59 males, as determined in 25 samples) were deposited in round plastic containers (13 cm diameter) with previously sterilised coarse sand (530 g) or coconut coir fibres (sterilised; 35 g) and 500 ml sterilised (chlorine-free) water (Figure 3). About 3 hours later (when all nematodes had moved into the substrate), the water was decanted and the containers covered and stored for eight weeks so that the nematodes could reach their sexual maturity, mate and deposit eggs. They were kept in a climate-controlled room at 27±2°C and 85% RH. Every week, condensation water droplets were removed from the containers with cotton tissue to prevent premature hatching of nematodes eggs.

### 2.3 Assessment of the yield of preparasitic juveniles

After eight weeks of storage, 20 containers were randomly selected from each of the coarse sand and coconut fibre groups. These containers were then flooded with 500 ml sterilised water to induce eclosion of the eggs and the emergence of infective J2 from the substrate. Fourteen hours after flooding the cultures (overnight), the water was decanted and the abundance of J2 and eggs in the water calculated by volumetric dilution [18]. Average abundance of J2 was assessed for a total of 320 containers for each of the substrates.

The impact of longer storage on the yield of infectious nematodes was also evaluated. The mean abundance of preparasitic nematodes was assessed 3-5 months after storage of J4s. The same procedures as described above were used.

All experiments were carried out in the Edward Platzer Centre for Integrated Malaria Vector Management (University of Abomey-Calavi, Benin), where a minimum of 150 million preparasitic *R. iyengari* are produced monthly for use against the larvae of malaria mosquitoes.

### 2.4 Statistical analysis

Mann–Whitney U tests were performed to determine whether there was a significant difference between the number of preparasitic nematodes on coconut coir fibres and on coarse sand substrates. Statistical analyses were performed with SPSS statistics package version 16.0.

## 3 Results

**Figure 4.**
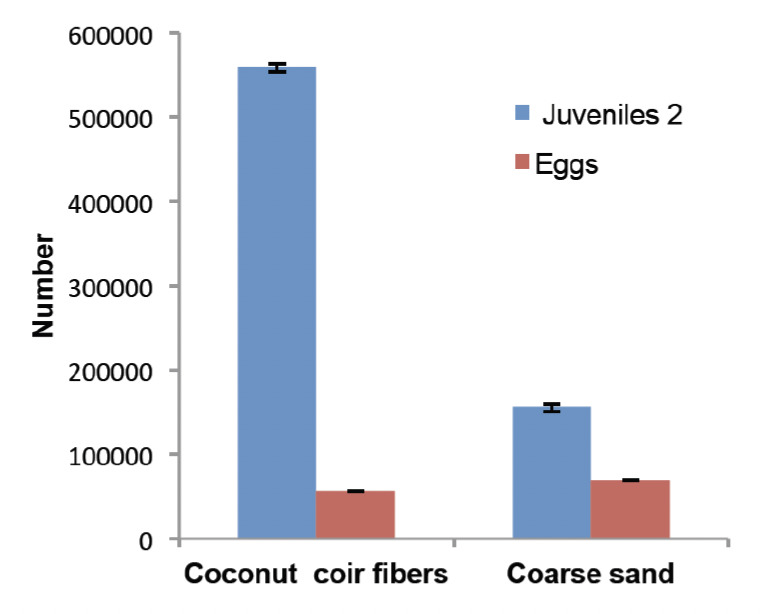
Average (± SE) abundance of infective preparasites and eggs obtained per container after 2 months storage of post-parasitic nematodes. (*n*=320 for each of the substrates)

Coconut coir fibres yielded 3.4 times more infectious nematodes (J2) than coarse sand as determined in 320 samples for each substrate (*P*<0.001) (Figure 4). The average abundance of eggs (which did not yet hatch 14 h after flooding) per container was significantly lower in coconut fibres (55,906±707) than in coarse sand (70,641±888) (*P*<0.001) (Figure 4).

**Figure 5.**
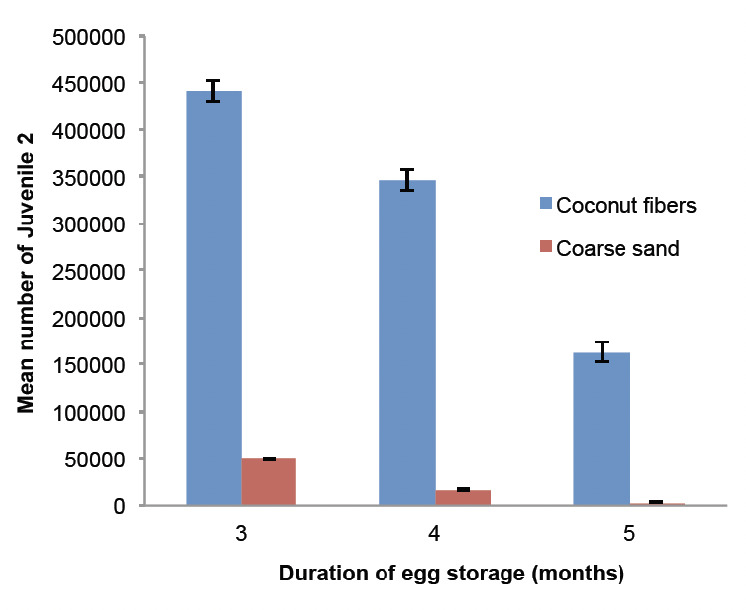
Impact of long-term storage on the yield (average ± SE) of infectious preparasites. (*n*=40 for each column)

Long-term storage data revealed that coconut fibres yielded significantly (*P*<0.001) more J2 than coarse sand. For coconut fibres, the mean number of J2 slowly decreased from 442,180±9322 (3 months storage) to 163,632±12,416 per container (5 months storage). On coarse sand, this figure, which initially was relatively low, decreased from 49,812±1200 J2 (3 months storage) to 3046±229 J2 per container (5 months storage) (Figure 5).

## 4 Discussion

Petersen *et al.* [18] established the first mass rearing facility for mermithid nematodes in the USA. Later, Perez-Pacheco *et al.* [19] developed a modified mass rearing procedure for *R. iyengari* for malaria mosquito control in Mexico. Both groups used coarse sand as a substrate for maturation of post-parasitic nematodes. In Benin, the mermithid production technology is based primarily on that used in Mexico, but we obtained lower juvenile 2 yields [12,19,20]. Relative air humidity conditions in the nematode storage room in Mexico were not published, but the temperature was reported to be 25±2°C [19]. In the storage room in Benin, the temperature was 27±2°C with 85% mean relative air humidity; these climatic conditions resulted in formation of water condensation in our nematode storage containers.

Cupello *et al.* [23] indicated earlier that free water formation in containers probably caused premature hatching of J2, thus reducing the yield of J2 when the containers were flooded. We therefore believe that the relatively low yield of infectious nematodes we obtained with coarse sand was attributable to the climatic conditions in our rearing centre. Substitution of coconut coir fibres for coarse sand resulted in a 3.4-fold increase in J2 yield. This yield level is satisfactory and enables the Benin production facility to produce a minimum of 150 million J2 per month. This productivity is similar to that obtained in Mexico where coarse sand is used [12]. The significant increase of J2 productivity with coconut fibres might be explained in part by the greater absorptive capacity of the coconut coir fibres for condensate as it occurs in the containers. Subsequently, eggs do not hatch after they mature, resulting in higher accumulation of unhatched eggs and thereby greater yield of J2 during 8 weeks of storage on coconut coir fibres.

Further study on the impact of long-term storage on the yield of infectious nematodes revealed that coconut fibres yielded also more J2 than coarse sand after 5 months storage. Overall, the coconut coir fibre technique is therefore more suitable than coarse sand in the high humid conditions of southern Benin.

Coconut coir fibres have several other advantages. The product is very light compared with sand, and will likely not damage the nematodes and eggs during transportation. Coarse sand is composed of small stone particles and the nematodes live and oviposit in the interstices. During transportation of the containers from the rearing centre to the larval control intervention sites these may crush the fragile nematodes and eggs due to movements. Vibration in the culture containers containing sand was a probable cause of nematode and egg loss in transportation trials by Petersen and Levy [23].

After setting up a rearing facility, the primary cost involved in the large-scale production of *R. iyengari* are the salaries of technical staff, maintenance of a blood meal source (chickens), and food for larval and adult mosquitoes (fish food and sugar, respectively). Coconut coir fibres are inexpensive and readily available in all coastal countries of West Africa. Three technicians can produce up to 150 million preparasitic nematodes monthly. To suppress larval malaria mosquito in stagnant breeding sites, 2,000-3,000 infectious nematodes (J2) per m^2^ are required [12,15,16]. With this spraying density, the current monthly nematode production is sufficient to suppress malaria vectors in 50,000-75,000 m^2^ of breeding sites. In West Africa, where salaries are relatively low, large-scale production of this insect parasitic nematode using coconut coir fibres offers thus an affordable alternative for malaria vector control.

## 5 Conclusions

The use of coconut coir fibres as a nematode substrate is a significant improvement for the cost-effective production of the insect parasitic nematode *R. iyengari* and has potential for facilitating the wider distribution of mermithid nematodes for use against malaria vectors in West Africa.
